# Population History and Altitude-Related Adaptation in the Sherpa

**DOI:** 10.3389/fphys.2019.01116

**Published:** 2019-08-28

**Authors:** Sushil Bhandari, Gianpiero L. Cavalleri

**Affiliations:** Department of Molecular and Cellular Therapeutics, Royal College of Surgeons in Ireland, Dublin, Ireland

**Keywords:** **** Sherpa, Tibetan, Sherpa physiology, hypoxia adaptation, genetic selection, high altitude adaptation, natural selection

## Abstract

The first ascent of Mount Everest by Tenzing Norgay and Sir Edmund Hillary in 1953 brought global attention to the Sherpa people and human performance at altitude. The Sherpa inhabit the Khumbu Valley of Nepal, and are descendants of a population that has resided continuously on the Tibetan plateau for the past ∼25,000 to 40,000 years. The long exposure of the Sherpa to an inhospitable environment has driven genetic selection and produced distinct adaptive phenotypes. This review summarizes the population history of the Sherpa and their physiological and genetic adaptation to hypoxia. Genomic studies have identified robust signals of positive selection across *EPAS1*, *EGLN1*, and *PPARA*, that are associated with hemoglobin levels, which likely protect the Sherpa from altitude sickness. However, the biological underpinnings of other adaptive phenotypes such as birth weight and the increased reproductive success of Sherpa women are unknown. Further studies are required to identify additional signatures of selection and refine existing Sherpa-specific adaptive phenotypes to understand how genetic factors have underpinned adaptation in this population. By correlating known and emerging signals of genetic selection with adaptive phenotypes, we can further reveal hypoxia-related biological mechanisms of adaptation. Ultimately this work could provide valuable information regarding treatments of hypoxia-related illnesses including stroke, heart failure, lung disease and cancer.

## Introduction

The term “sher-pa” is the Tibetan for “eastern-people”. The Sherpa reside primarily in the Solukhumbu district of Nepal but there are also smaller settlements in the Tibet Autonomous Region of China. The Sherpa speak a Tibetan dialect, and they share similar cultural and religious practices with Tibetans. They are traditionally engaged in farming; cultivating barley, potatoes and rearing yak and sheep. Starting with the first Everest expeditions in the 1920’s, the Sherpa have become renowned for their ability as mountaineers and today they often aid and lead climbing expeditions in the Himalayas. Examples of their exceptional climbing feats include the first ascent of Mount Everest by Tenzing Norgay Sherpa, who accompanied Sir Edmund Hillary in the final stage of the 1953 expedition and Ang Rita Sherpa (known as “The Snow Leopard”) who, between 1983 and 1996, summited Everest ten times without the use of supplemental oxygen. The remarkable tolerance of the Sherpa to hypoxia has, over the last 60 years, been a focus of attention for the scientific community, in particular physiologists (Gilbert-Kawai et al., 2014).

The Sherpa are direct descendants of an ancestral population that has resided continuously on the Tibetan plateau for the past 25,000 to 40,000 years (Aldenderfer, 2011; Zhang et al., 2018). This long exposure to the evolutionary pressure presented by high altitude has driven physiological adaptation, which in turn has allowed the Sherpa to thrive. The adaptive physiological makeup of the Sherpa can inform on treatments for hypoxia-related illness including pulmonary, cardiac, neurological and renal disorders (Martin et al., 2013; Luks and Hackett, 2014; Gilbert-Kawai et al., 2015). Thus, studying the Sherpa at altitude offers a unique, “natural laboratory” that can provide insight to the molecular mechanisms of hypoxia.

An early paper on Sherpa physiology, published in 1965, suggested that the Sherpa have an efficient mechanism of oxygen utilization at the cellular level, allowing them to perform well under hypoxia (Lahiri and Milledge, 1965). Since then, our knowledge of Sherpa adaptation has grown, largely by comparing different physiological parameters between the Sherpa and people of lowland origin. With the development of high throughput DNA genotyping and sequencing platforms, genomic studies of indigenous high-altitude populations, including the Sherpa, have begun to emerge. These have provided insight into population history and genetic signatures of altitude-driven natural selection. In this review, we (1) summarize the population history of, (2) describe distinct adaptive phenotypes and (3) discuss signatures of selection, in the Sherpa. We highlight the need for further research connecting genetic factors to physiological adaptation in the Sherpa at extreme altitude.

## The Sherpa, a Recently Derived Tibetan Population

Stone tools used by early humans have been found at Nwya Devu in central Tibet at an altitude of 4,600 m. Dating to 30,000 to 40,000 years before present (YBP), these findings represent the earliest archeological record of human colonization of the Tibetan plateau (Zhang et al., 2018). Genetic studies have suggested that the ancestors of both the Sherpa and Tibetans diverged from a Han Chinese population and arrived on the Tibetan plateau from lowland East Asia around 40,000 years ago (Qi et al., 2013; Jeong et al., 2014).

The prevailing hypothesis is that, during the 16th century, the ancestors of the Sherpa migrated from Tibet to the Khumbu Valley of Nepal, driven by political and religious turmoil resulting from a Mongol invasion (Oppitz, 1974). The presence of Sherpa-specific mitochondrial DNA (mtDNA) lineages (Kang et al., 2013) in a Nepalese context, with an estimated age of less than 1,500 years and derived from Tibetans, further supports this hypothesis of a recent migration of the Sherpa to the Khumbu valley (Bhandari et al., 2015).

There is a long history of migration from the Tibetan plateau to Nepal. To illustrate, genomic analysis of human dental samples (dating to between 1,700 and 3,000 YBP) from a northern region of Nepal show strong affinity for contemporary Tibetans (Jeong et al., 2016). Analysis of both autosomal data (Lu et al., 2016; Gnecchi-Ruscone et al., 2017) and uniparental mtDNA and Y-chromosome markers (Bhandari et al., 2015) have shown the Sherpa and Tibetans to share relatively recent common ancestry. Tibetans also share recent common ancestry with other Nepalese populations including the Rai, Magar, Tamang, and Gurung (Cole et al., 2017). The Sherpa share more genetic affinity with these Tibeto-Burman speaking populations than with other Indo-Aryan populations of Nepal. However, the Sherpa are distinct from other Nepalese populations in that the Sherpa have elevated levels of runs of homozygosity (Cole et al., 2017), and illustrate very little or no admixture with Nepalese or South Asian populations (Cole et al., 2017). Thus, the Khumbu Valley Sherpa can be considered from the perspective of population genetics as a “bottlenecked” population recently derived from Tibetans.

## Comparative Physiological Studies Between Sherpa and Lowlanders

In 1952, Griffith Pugh conducted a series of pioneering physiological experiments on Mount Cho Oyo (at 8,188 m, 20 km west of Mount Everest) that suggested a superior work capacity of the Sherpa at high altitude (Pugh, 1962; Pugh et al., 1964). They also provided the scientific rationale for the hydration, nutrition and oxygen requirements for the first Everest summiting in 1953 (Milledge, 2002). Although the physiology of the Sherpa has been studied over the intervening 60 years, the scientific literature is limited in number, and most of the studies are based on small sample sizes. There are obvious challenges to studying the Sherpa; they reside in a remote region, at an altitude over 2,800 m, where altitude sickness is common for sojourners. Despite this, several remarkable findings have emerged and below we discuss specific phenotypes that may be linked to hypoxia-related genetic signals of selection reported to date. For a discussion of other hypoxia-related physiological parameters studied in Sherpa, such as ventilation, lung volume, exercise capacity and cerebral function (see Gilbert-Kawai et al., 2014; Table 1).

**TABLE 1:** Physiological parameters studied in Sherpa and lowlanders at altitude.

**Parameter(s)**	**Sherpa at high altitude**		**Lowlander at altitude (meter)**				**Reference(s)**
	**Sample size**	**Parameter value**	**Altitude**	**Sample size**	**Duration (days)**	**Parameter value**	
Heart rate while working at 900 kg⋅m/min-beats/min	1	162	5,800	2	240	122	Pugh, 1962; Pugh et al., 1964
Lung diffusion capacity for oxygen-ml/min	1	97	5,800	2	240	52.5	Pugh, 1962
Basal metabolic rate, kcal/m^2^ h	3	46.1 ± 1.0	5,800	8	240	41.1 ± 3.6	Gill and Pugh, 1964
10 different physiological parameters; measured, to test oxygen utilization at the cellular level	^4^	efficiently used O_2_	4,880	3	60	less efficient to use O_2_	Lahiri and Milledge, 1965
Heart rate (while work rate at 1,265 kg-m/min)- beats/min	4	198	4,880	2	63	146	Lahiri et al., 1967
Partial pressure of carbon dioxide in the arterial blood, mm Hg	^4^	28.6	4,880	5	60	25.9	Lahiri and Milledge, 1967
Hemoglobin level in Tibetans living at 3658 m in Nepal; g/l00 ml	52	Male; 16.8 ± 1.4; Female: 14.5 ± 0.7				–	Adams and Shresta, 1974
Hemoglobin level in Tibetans living at 4000 m in Nepal; g/l00 ml	51	Male; 17.0 ± 1.25; Female:15.3 ± 0.8				–	Adams and Strang, 1975
Ratio of 2, 3 diphosphoglycerate and hemoglobin	7	0.9	3,900	2	30	1.26	Morpurgo et al., 1976
Mean oxygen half saturation of hemoglobin	7	27.3 ± 1.8	3,500	7	120	28.2 ± 1.3	Samaja et al., 1979
Arterial oxygen saturation (SaO_2_)	10	88 ± 0.74	4,243	25	12	85.6 ± 1.0	Hackett et al., 1980
Body weight changes- Mean weight loss (kg)	4	constant	5,400	13	25	1.9 to 4	Boyer and Blume, 1984
Hypoxic ventilatory response (HVR)-end-tidal PO_2_, 40 Torr			6,300	9	25	21.2 ± 5.4	Schoene et al., 1984
Partial pressure of oxygen in arterial blood (Torr)	6	34.5 ± 3.2	5,400	9	–	41.0 ± 3.3	Santolaya et al., 1989
Partial pressure of carbondioxide in arterial blood (Torr)	6	27.5 ± 2.2	5,400	9	–	20.0 ± 2.8	Sutton et al., 1988
Hemoglobin oxygen affinity values	14	29.8 ± 1.9	–	1	–	19	Winslow et al., 1989
Resting glucose appearance rate at sea level (1.79 ± 0.02) mg.kg−l.min-1	–	–	4,300	7	21	3.59 ± 0.08	Brooks et al., 1991
HVR shape parameter A, (mean ± SE)	27	121 ± 17	3,658	30	9 ± 1 year	81 ± 10	Zhuang et al., 1993
Resting mean pulmonary arterial pressure SE mmHg	5	15 ± 1		22		28 ± 2	Groves et al., 1993
Glucose metabolic rates of myocardial regions	6	0.32 ± 0.05	226	6		0.20 ±0 04	Holden et al., 1995
Brain glucose metabolic rates	^6^	0.71		6	19	0.73	Hochachka et al., 1996b
Signs of mild cortical atrophy	7	Seen in 1		21		Seen in 13	Garrido et al., 1996
Partial pressure of carbon dioxide, mm Hg	5	28.8 ± 1.2	3,400	4	40	22.0 ± 0.4	Samaja et al., 1997
Mean arterial blood pressure, mm Hg	9	83 ± 6	4,243	10	7	94 ± 7	Jansen et al., 2000
Forced expiratory volume of adult male (%)	146	110(107−114)	3,840	103		103.8 (100.4-107.3)	Havryk et al., 2002
Heart Rate (beats min–1) means ± S.D.	7	167 ± 10	5,050	10	28	149 ± 7	Marconi et al., 2004
Carried loads of their body weight (mean ± SD)	96	93 ± 36%	2,880	10		75%	Bastien et al., 2005a, b, 2016
Arterial oxygen saturation (SaO_2_) or (SpO_2_)	–	–	5,620	lower SaO*_2_* in Han than Tibetans			Wu, 1990; Wu and Kayser, 2006
Arterial oxygen saturation, %	10	88 ± 3	40	10		97 ± 2	Jansen et al., 2007
Statistically significant gender specific differences in SpO_2_	Adult Tibetan female show higher SpO_2_ value than male						Weitz and Garruto, 2007
Serum angiotension-converling enzyme activity, IU/L/37°C	105	14.5 ± 0.4	1,300	111		14.7 ± 0.4	Droma et al., 2008
Mean arterial oxygen content at 8,400 m (26% lower than at 7100 m)	–	–	8,400	4	–	145.8 ml per L	Grocott et al., 2009
Muscle phosphocreatine recovery halftime- PCr_tl/2_ (s)	7	22.2 ± 1.6	50	7	–	16.1 ± 1.1	Edwards et al., 2010
Radial arterial plasma NO_2_^–^(nmol I^–1^)	–	–	4,559	26	4	263.6 ± 61.2	Bailey et al., 2010
Middle cerebral artery diameter [at 6,400 m = 6.66 mm]	–	–	7,950	5	71	9.34 mm	Wilson et al., 2011
Flow-mediated dilatation (FMD)-shear rate	12	24490 ± 7230	5,050	12	14	14802 ± 5306	Lewis et al., 2014
Arterial oxygen saturation (mean ± SE)	13	86 ± 1	5,050	13	9	83 ± 2	Faoro et al., 2014
Hemoglobin level ml. min(−l). mmHg(−l)	13	61 ± 4	5,050	13	9	37 ± 2	Faoro et al., 2014
Lung diffusing capacities	13	226 ± 18	5,050	13	9	153 ± 9	Faoro et al., 2014
Systolic pulmonary artery pressure	95	29.4 ± 5.5	13	64	–	23.6 ± 4.8	Bruno et al., 2014
Left ventricular untwisting velocity, °/s	11	−93 ± 31	5,050	9	13	−153 ± 38	Stembridge et al., 2014
Right ventricular isovolumic ralaxation time, ms	11	64 ± 20	5,050	9	13	78 ± 14	Stembridge et al., 2015
No significant differences of dietary nitrate supplementation on AMS score	–	–	4,559	28	7	*p* = 0.29, *p* = 0.47	Cumpstey et al., 2017
Arterial oxygen saturation (%, 95% CI of Mean)	–	–	5,300	11	13	73.0 (70.3–75.5)	Luks et al., 2017
Relative *PPARA* mRNA expression of muscles tissues	15	0.5158	5,300	10	19	1.0045	Horscroft et al., 2017
Post reproductive, Tibetan women (*n*=959)-Hemoglobin concentration, gm/dl	***–***	13.8			–	–	Cho et al., 2017
Increase in nocturnal time course of blood oxygen saturation level at rest	–	–	3,050	10	21	94.5% (91-97)	Tannheimer et al., 2017
FMD unchanged (in rest and maximal exercise), at low and high altitude	–	–	3,800	9	7	(6.3 ± 1.3)%	Tymko et al., 2017a
Brachial artery blood flow [at Sea level- (142.7 ± 30.6)], ml/min	–	–	5,050	14	21	53.1 ± 11.1	Tymko et al., 2017b
Number of circulating microparticles in blood (CD 66b+)/μ1 (21 ± 4) Sea level		–	3,800	10	3	74 ± 17	Tremblay et al., 2017
Birth-weight (kg) in Tibetans & Han; at 3,000–4,000 m altitude	100	3.14 (3.06, 3.22)	<4,000	100		2.61 (2.34, 2.88)	Moore et al., 2001
Case report of a 32 week gestation Sherpa at 5160 m and her data after 10 month postpartum	No apparent maternal, fetal or neonatal complications					–	Davenport et al., 2018
Arterial oxygen pressure (PaO_2_; mm Hg)	–	–	4,100	8	50	54 ±1.2	Lundby et al., 2018
Prefatigue, maximal voluntary contraction torque, N. m	9	50.1 ± 11.3	5,050	9	–	–	Ruggiero and Mcneil, 2018
Maximal voluntary contractile force (kg)	10	44.3 ± 14.1	5,050	12	10	58.2 ± 8.1	Ruggiero et al., 2018
Brachial artery flow-mediated dilation (FMD)	12	5.8 ± 2.8%	5,050	22	10	3.8 ± 2.8%	Tremblay et al., 2018
Resting posterior cerebral artery velocity	–	–	4.240	10	13	43 cm/s	Leacy et al., 2018
Lowland origin; Female SpO_2_; Mean (SD), (%)[95.2 (1.2); at 600 m]	–	–	3,500	20	1	76.7 (5.6)	Burtscher et al., 2018
Partial pressure of arterial carbon dioxide. mmHg	11	32.1 ±2.5	5,050	21	21	30.0 ±1.9	Willie et al., 2018
Peripheral oxygen saturation in female [at 600 m; 96.9 (1.0)] Mean (SD) %	–	–	3,840	20	1	86.5 (6.5)	Burtscher et al., 2019
SpO_2_ (%) [at Sea Level (244 m) is 98 ± 1]	–	–	3.800	12	10	89.1 ± 3	Stembridge et al., 2019
Free cysteine and plasma total free thiol concentrations	–	–	4,559	4	Elevated at 4,559 m than at 50 m		Cumpstey et al., 2019
Sublingual capillary total vessel density [at Sea Level; 18.81 ± 3.92 mm mm^–2^	–	–	7,042	10	21	21.25 ±2.27	Hilty et al., 2019
Sympathetic nerve activity, burst frequency (bursts min^–1^)	8	22 ± 11	5,050	14	20	30 ± 9	Simpson et al., 2019

### Hemoglobin Concentration

The hypoxic challenge presented by high altitude drives changes in hemoglobin concentration. Elevated hemoglobin levels (≥19 g/dl in females; and ≥21 g/dl in males) resulting from hypoxia can lead to chronic mountain sickness (Leon-Velarde et al., 2005). Relative to lowland controls, the literature suggests the Sherpa display lower hemoglobin concentrations at high altitude (Beall and Reichsman, 1984; Wu et al., 2013; Bhandari et al., 2016). Sherpa women with lower hemoglobin concentrations (13.8 g/dl ± 1.3 g/dl) are reported to have better reproductive outcomes (Beall et al., 1997, 2004; Cho et al., 2017). Increased exercise capacity has been reported in Tibetan males with a low erythropoietic response (Simonson et al., 2015). It is yet to be determined whether the lower hemoglobin concentration observed in Sherpa is due to a blunted erythropoietic response or to some other physiological parameters that impact hemoglobin concentration.

### Nitric Oxide Concentration

Nitric oxide acts as a vasodilator and is believed to protect against pulmonary hypertension at high altitude (Busch et al., 2001). It also plays a role in haematocrit regulation by controlling blood viscosity (Ashmore et al., 2014). Serum nitric oxide levels have been reported as reduced in the Sherpa relative to lowlanders (Droma et al., 2006), and a recent study reported no differences in circulating nitric oxide metabolites [N-nitrosamine (RNNO), S-nitrosothiol, nitrate, or nitrite concentrations] between Sherpa and lowlanders at both low and high altitude (Horscroft et al., 2017). However, a non-synonymous variant (rs549340789) in NOS1 (nitric oxide synthases 1) has been identified as under positive selection in the Sherpa (Zhang et al., 2017). Thus, it seems that nitric oxide may play an important role in hypoxic adaptation (Erzurum et al., 2007; Beall et al., 2012), but the exact mechanisms remain poorly understood.

### Microcirculation

Lowlanders exhibit, in a hypoxic environment, reduced sublingual microcirculatory blood flow (Martin et al., 2009). However, the Sherpa maintain sublingual capillary densities and microcirculatory blood flow (Gilbert-Kawai et al., 2017) at altitude. Compared to mountaineers of European-ancestry, during an expedition to Mount Everest, Sherpa exhibit elevated basal levels of angiogenic elements including vascular endothelial growth factor A (VEGF-A), interleukins (IL-8) and lymphangiogenic factors (VEGF-C and D), which likely facilitate increased microcirculatory flow (Patitucci and Lugrin, 2009). The Sherpa display an elevated oxygen unloading rate, and increased myogenic activity relative to lowlanders, further supporting higher peripheral microcirculatory perfusion (Davies et al., 2018). Following a defined period of induced leg occlusion and muscle ischemia, the Sherpa are reported to display increased blood flow velocity, relative to lowlanders (Schneider et al., 2001). This is likely due to differences in conduit vessel function. Thus, the Sherpa appear to exhibit distinct microcirculation patterns, which might facilitate increased tissue oxygen transfer to overcome hypoxia.

### Pulmonary and Cardiac Physiology

The Sherpa have greater spirometry values, forced expiratory volumes and forced vital capacity relative to lowlanders at high altitude (Pugh, 1962; Havryk et al., 2002). Lowlanders often experience apnea-induced brady-arrhythmias at high altitude, while Sherpa typically do not (Busch et al., 2017). The Sherpa display lower pulmonary vascular resistance and smaller left ventricular end-diastolic volume (Stembridge et al., 2014). However, the mechanism by which this reduced myocardial relaxation impacts on the exercise capacity of the Sherpa is unclear (Stembridge et al., 2015).

Evidence suggests a shift in cardiac substrate preference, from fat to glucose, in Sherpa relative to lowland controls (Holden et al., 1995). Some patients with heart failure display a reduction of the myocardial PCr to ATP ratio (Neubauer et al., 1997). Lowlanders returning from high altitude also display a significant decrease in myocardial PCr/ATP ratio (Holloway et al., 2011), but this ratio remains steady in the Sherpa (Hochachka et al., 1996a).

### Skeletal Muscle

Sherpa muscle contains a significantly greater number of capillaries per cross-sectional area, in comparison to lowlanders (Kayser et al., 1991). Sherpa also display a reduced mitochondrial content, but their muscle is somehow maximizing the oxygen consumption to mitochondrial volume ratio (Kayser et al., 1991; Horscroft et al., 2017). Under hypoxia, Sherpa skeletal muscle prefers carbohydrate over fatty acids as a metabolic substrate (Murray, 2009). Sherpa muscle maintains fatty acid oxidation relative to lowlanders at high altitude. Incomplete fatty acid oxidation results in production of byproducts such as acylcarnitines and reactive oxygen species. Acylcarnitines and markers of oxidative stress (e.g., reduced/oxidized glutathione and methionine sulfoxide) are increased in lowlander muscle relative to the Sherpa (Gelfi et al., 2004; Horscroft et al., 2017). However, oxidative damage in lowlanders was reduced to levels comparable with the Sherpa, where acclimatization has taken place (Janocha et al., 2017). Lactate dehydrogenase activity is elevated in Sherpa muscle (Allen et al., 1997; Horscroft et al., 2017), indicating greater capacity for anaerobic lactate production. With increasing altitude, lowlanders experience a gradual reduction in phosphocreatine (PCr) and ATP levels (Levett et al., 2015). But the Sherpa maintain PCr and ATP levels at altitude (Horscroft et al., 2017). Thus, the superior muscle energetics displayed by the Sherpa is probably the result of adaptation at the metabolic level.

### Birth Weight

Women of European and Han Chinese ancestry exhibit reduced birth weights following gestation at high altitude, quantified at 100 *g* reduction for every 1,000 m elevation (Moore, 2003; Julian et al., 2009; Moore et al., 2011). The Sherpa (and Tibetans), however, maintain normal birth weight at both low (1,330 m) and high (3,930 m) altitude (Smith, 1997; Moore et al., 2001). Genes including *PPARA* are expressed in the placenta (Barak et al., 2008) and have been shown to influence female reproductive function (Bogacka et al., 2015). HIFs play a critical role in mammalian embryo and placental development (Dunwoodie, 2009; Pringle et al., 2009). *EPAS1* expression appears reduced in umbilical endothelial cells and placentas of Tibetan women (Peng et al., 2017). Intronic variants in *CCDC141* have been shown in Tibetan and Sherpa women to associate with the number of live births, and the same locus also shows evidence of positive selection (Jeong et al., 2018). The increased reproductive success of the Sherpa is therefore likely to be, at least in part, due to cardiac-related traits (Jeong et al., 2018) and placental adaptation (Burton et al., 2016). Further studies are required to understand the molecular mechanisms by which the Sherpa maintain normal intrauterine growth at altitude.

In summary, the Sherpa display distinct physiological responses to hypoxia that contrast to lowlanders at high altitude (Table 1). These are presumably the result of exposure over many generations to the hypoxia-related selective pressure presented by the Tibetan plateau. Indeed, some examples have already emerged of specific genetic signatures of selection associating with distinct adapative traits (Simonson, 2015; Moore, 2017).

## Signatures of Altitude-Related Genetic Selection in the Sherpa

With developments in sequencing and genotyping technology over the past decade, it has become possible to identify population-specific signatures of selection for adaptation across the human genome. There are now several complementary genomic tests available for detecting genetic selection (Scheinfeldt and Tishkoff, 2013) and the application of these tests to data from indigenous high-altitude people including the Sherpa have identified numerous and remarkable genetic signals of selection. Here, we focus on the three most robust signals of selection detected to date in the Sherpa: *EPAS1*, *EGLN1*, and *PPARA* (Table 2).

**TABLE 2:** A summary of genetic adaptations reported in the Sherpa, and replication in other population(s) or species.

**Genes name(s)**	**Sherpa**		**Other population(s) or species**	**Reference(s)**
	**Sample Size**	**Reference(s)**		
ACE	105	Droma et al., 2008	Elite European descent athletes	Montgomery et al., 1998; Jones et al., 2002
*HIF-la*	20	Suzuki et al., 2003	–	–
*eNOS*	105	Droma et al., 2006		
*EPAS1*	105	Hanaoka et al., 2012	Tibetan	Beall et al., 2010; Simonson et al., 2010; Yi et al., 2010; Bigham et al., 2010; Peng et al., 2011; Wang et al., 2011; Xu et al., 2011
	51	Jeong et al., 2014	Deedu Mongolian	Xing et al., 2013
	582	Bhandari et al., 2016	Denisovan	Huerta-Sánchez et al., 2014
3.4 kb Copy Number Deletion-80 kb downstream of *EPAS1*	582	Bhandari et al., 2016	Tibetan	Lou et al., 2015
*EGLN1*	51	Jeong et al., 2014	Tibetan	Lorenzo, 2010; Simonson et al., 2010; Yi et al., 2010; Xiang et al., 2013; Lorenzo et al., 2014
	582	Bhandari et al., 2016	Andean	Bigham et al., 2009; Bigham et al., 2010
	111	Zhang et al., 2017	Daghestani	Pagani et al., 2012
*PPARA*	15	Horscroft et al., 2017	Tibetan	Simonson et al., 2010; Peng et al., 2011
		Kinota et al., 2018	(Amhara and Omotic) Ethiopian	Scheinfeldt et al., 2012
*HYOUI/HMBS*	51	Jeong et al., 2014	–	–
*EPAS1*, *EGLN1*, *DLG1*, *MARCH8*, *CDCA7L*, *HEATR5B*, *EDAR*, *ZNF644*, *TTC24*, *TMEM247*, *OXR1*, *ALDH31*	111	Zhang et al., 2017	–	–
*NOS1*	111	Zhang et al., 2017	Tibetan *(GCH1)*, Andeans (*NOS2*)	Bigham et al., 2009; [opetwcitep]B20,B59[clotwcitep]Bigham et al., 2010; He et al., 2018
*ANGPT1*	111	Zhang et al., 2017	Tibetan and grey wolves of TAR, China	Jeansson et al., 2011; Wang et al., 2011
*EPAS1*, *EGLN1*, *RP11-384F7.2 AC068633.1*, *ZNF53 2*, *HLA-DOB1/HLA-DPB1*	10	Arciero et al., 2018	–	–
*ANKH*	10	Arciero et al., 2018	Pigs of TAR, China	Ai et al., 2014
*GRB2*	10	Arciero et al., 2018	Tibetans	Li et al., 2016
Polygeneic Adaptation {Gene subnetworks like the nested integrin associated pathways (i.e., Integrin β-1, Integral α6−β4 and Integrin involved in angiogenesis),CMYB and C-MYC transcription factor pathways}	31	Gnecchi-Ruscone et al., 2017, 2018	Tibetans	Gnecchi-Ruscone et al., 2018
*EPAS1, EGLN1.CCDC141,PAPOLA, VRK1, C6orf195, CTBP2, TEX36, EDRF1*	103	Jeong et al., 2018	Tibetans	Jeong et al., 2018

### Endothelial PAS Domain-Containing Protein 1 (EPAS1)

One of the earliest signals for altitude-related adaptation to emerge from genomic selection studies was *EPAS1.* Initially discovered in Tibetans (Beall et al., 2010), the *EPAS1* signal has been replicated in multiple other Tibetan populations (Bigham et al., 2010; Simonson et al., 2010; Yi et al., 2010; Peng et al., 2011; Wang et al., 2011; Xu et al., 2011) as well as the Sherpa (Hanaoka et al., 2012; Jeong et al., 2014; Bhandari et al., 2016). The selected *EPAS1* haplotype is associated with lowered hemoglobin concentrations (Beall et al., 2010). Remarkably, it seems the adaptive *EPAS1* haplotype likely descends from an introgression event with the Denisovan people, an extinct species of archaic humans (Huerta-Sánchez et al., 2014; Hu et al., 2017). A 3.4 kb copy number deletion, downstream of *EPAS1*, is elevated in frequency, in Tibetans and Sherpas relative to lowland controls (Lou et al., 2015). This deletion is in strong linkage disequilibrium with the previously reported (Beall et al., 2010) *EPAS1* haplotype and has also been associated with lower hemoglobin levels. The actual functional *EPAS1* variant(s) that are conferring advantage in relation to hypoxic adaptation remain unknown. However, the intronic and intergenic location of the selected variants would be consistent with a role in HIF-related transcriptional regulation.

*EPAS1* encodes the HIF2 alpha subunit of HIF2. The postnatal deletion of *EPAS1* in adult mice causes anaemia (Gruber et al., 2007). Some cases of erythrocytosis are caused by missense mutations (e.g., G536W) in *EPAS1* (Percy et al., 2008). Mice carrying the *EPAS1* G536W mutation display excessive erythrocytosis and pulmonary hypertension (Tan et al., 2013). Another study in heterozygous *EPAS1* knockout mice reported a blunted physiological response to chronic hypoxia (Peng et al., 2017). Further *in-vivo* and *in-vitro* studies are necessary to understand how the adaptive version of the *EPAS1* gene is shaping human adaptation to altitude.

### Egl-9 Family Hypoxia Inducible Factor 1 (EGLN1)

Another high altitude genetic selection signal to emerge from early studies on Tibetans was *EGLN1* (Simonson et al., 2010; Yi et al., 2010). Similar to *EPAS1*, this signal was later demonstrated in the Sherpa (Jeong et al., 2014). Two functional *EGLN1* mutations (rs12097901, D4E, and rs186996510, S127C) appear to be driving the selection signal and are present in both Sherpa (Bhandari et al., 2016) and Tibetans (Lorenzo, 2010; Xiang et al., 2013; Lorenzo et al., 2014). Whether the mode of action of these two mutations is via gain of function (Lorenzo et al., 2014) or loss of function (Song et al., 2014) remains unclear.

*EGLN1* encodes proline hydroxylase 2 (PHD2), an isoform of HIF prolyl-hydroxylase. Homozygous knockout PHD2 mice are unviable and die at the embryonic stage due to severe placental defects (Takeda et al., 2006). Knockout mice with PHD2 disruption targeted to specific organs including the liver, heart, kidney and lung develop excessive vascular growth (Takeda et al., 2007). Adult mice deficient for PHD2 display excessive erythrocytosis (Takeda et al., 2008) and heterozygous PHD2 mice have an increased ventilatory sensitivity to hypoxia and carotid body hyperplasia (Bishop et al., 2013).

### Peroxisome Proliferator-Activated Nuclear Receptor A (PPARA)

*PPARA* encodes PPARα, a transcriptional regulator of fatty acid oxidation in liver, heart and muscle (Gilde and Van Bilsen, 2003). *PPARA* has tissue-specific expression and, under hypoxic conditions, is downregulated by HIFs (Narravula and Colgan, 2001). Positive selection across the *PPARA* gene has been reported in Tibetans (Simonson et al., 2010) and Sherpa (Horscroft et al., 2017), and the selected *PPARA* SNPs correlate with reduced hemoglobin levels (Simonson et al., 2010). Sherpa carriers of the positively selected *PPARA* alleles switch to more efficient fuels such as glucose and display decreased muscular fatty acid oxidation (Horscroft et al., 2017). Most of the *PPARA* SNPs reported to be under selection appear to be non-coding variants (Kinota et al., 2018). It is unclear if these variants directly affect transcriptional regulation or are linked with functional variants in other genes or nearby inter-genic regions.

## Conclusion

The Sherpa show remarkable performance in the hypoxic environment presented by high altitude. Comparative physiological studies have suggested numerous distinct, adaptive phenotypes in the Sherpa including advantageous levels of hemoglobin, oxygen saturation and birth weight, and the elevated reproductive success of Sherpa women. Genomic studies have identified robust signals of positive selection across genes including *EPAS1*, *EGLN1* and *PPARA*. All three of these signals of genetic selection have been shown to correlate with advantageous levels of hemoglobin. However, Sherpa-specific signals of genetic selection have also been reported, suggesting that whilst some of the genetic basis for adaptation in the Sherpa is shared with Tibetans, there may be features unique to the Sherpa, which could in turn explain distinct Sherpa phenotypes. Collectively, this illustrates how the outstanding physiological performance of the Sherpa at altitude is, at least in part, a result of hypoxia driven genetic selection spanning the ∼35,000 years of seasonal migration on the Himalayan plateau. Further comparative physiological studies are required to refine existing, and identify additional adaptive phenotypes, in particular those that are specific to the Sherpa. By correlating these phenotypes with known and emerging signals of genetic selection, we can shed light on biological mechanisms of Sherpa hypoxic adaptation. Ultimately this work can inform on treatments of hypoxia-related illness including pulmonary, cardiac, neurological and renal disorders.

## Author Contributions

Both authors drafted, edited, and approved the final version of the manuscript.

## Conflict of Interest Statement

The authors declare that the research was conducted in the absence of any commercial or financial relationships that could be construed as a potential conflict of interest.
